# SnRK2.10 kinase differentially modulates expression of hub WRKY transcription factors genes under salinity and oxidative stress in *Arabidopsis thaliana*

**DOI:** 10.3389/fpls.2023.1135240

**Published:** 2023-08-09

**Authors:** Julia Rachowka, Anna Anielska-Mazur, Maria Bucholc, Krystyna Stephenson, Anna Kulik

**Affiliations:** Institute of Biochemistry and Biophysics, Polish Academy of Sciences, Warsaw, Poland

**Keywords:** SnRK2, stress signaling, salinity, oxidative stress, WRKY TFs

## Abstract

In nature, all living organisms must continuously sense their surroundings and react to the occurring changes. In the cell, the information about these changes is transmitted to all cellular compartments, including the nucleus, by multiple phosphorylation cascades. Sucrose Non-Fermenting 1 Related Protein Kinases (SnRK2s) are plant-specific enzymes widely distributed across the plant kingdom and key players controlling abscisic acid (ABA)-dependent and ABA-independent signaling pathways in the plant response to osmotic stress and salinity. The main deleterious effects of salinity comprise water deficiency stress, disturbances in ion balance, and the accompanying appearance of oxidative stress. The reactive oxygen species (ROS) generated at the early stages of salt stress are involved in triggering intracellular signaling required for the fast stress response and modulation of gene expression. Here we established in *Arabidopsis thaliana* that salt stress or induction of ROS accumulation by treatment of plants with H_2_O_2_ or methyl viologen (MV) induces the expression of several genes encoding transcription factors (TFs) from the WRKY DNA-Binding Protein (WRKY) family. Their induction by salinity was dependent on SnRK2.10, an ABA non-activated kinase, as it was strongly reduced in *snrk2.10* mutants. The effect of ROS was clearly dependent on their source. Following the H_2_O_2_ treatment, SnRK2.10 was activated in wild-type (wt) plants and the induction of the WRKY TFs expression was only moderate and was enhanced in *snrk2.10* lines. In contrast, MV did not activate SnRK2.10 and the WRKY induction was very strong and was similar in wt and *snrk2.10* plants. A bioinformatic analysis indicated that the *WRKY33, WRKY40, WRKY46*, and *WRKY75* transcription factors have a similar target range comprising numerous stress-responsive protein kinases. Our results indicate that the stress-related functioning of SnRK2.10 is fine-tuned by the source and intracellular distribution of ROS and the co-occurrence of other stress factors.

## Introduction

The environment is highly dynamic and undergoes constant changes. The fluctuating environmental conditions that have a negative impact on plant functioning are considered stress factors (Srivastava et al., 2021). Salinity is one of the most common environmental factors limiting plant productivity and affecting agricultural yield (Acosta-Motos et al., 2017). It is estimated that globally about 20% of irrigated land is affected by salinity and therefore unsuitable for agriculture, and by 2050 this fraction will increase to 50% (Srivastava et al., 2021). The main deleterious effects of salinity are the reduction of water potential and the appearance of water deficiency stress, and disturbances in ion balance. As a result, salt stress limits plant growth, inhibits photosynthesis, affects redox homeostasis, modulates antioxidant metabolism, the uptake and balance of mineral nutrients, and accumulation of osmolytes (for review see Yang and Guo, 2018; Hasanuzzaman et al., 2021; Srivastava et al., 2021). A proper recognition of the stress conditions, triggering adequate signaling pathways and metabolic adjustment are critical for the optimization of plant growth, reproduction, and survival under challenging conditions.

Stress signals are recognized and transmitted to diverse cellular compartments by specialized signaling pathways in which protein kinases and phosphatases are key components. Among the protein kinases involved in stress and ABA signal transduction, Sucrose non-fermenting 1-Related protein Kinases (SnRKs) play an important role. The SnRKs are classified into three subfamilies: SnRK1, SnRK2, and SnRK3. In this study, we focus on the SnRK2 subfamily, which are plant-specific Ser/Thr protein kinases. They have been identified in *Arabidopsis thaliana* (Boudsocq et al., 2004) and in other species such as rice, sorghum, maize, tobacco, wheat, soybean, fava bean, potato, and algae (for review see Kulik et al., 2011 and Shinozawa et al., 2019; Kamyiama et al., 2021). The SnRK2 subfamily has been divided into three groups, based on their reaction to the phytohormone abscisic acid (ABA): group 1 – kinases not activated by ABA, group 2 – weakly activated by this hormone, and group 3 - kinases strongly activated by ABA. All SnRK2s are activated rapidly in response to osmotic stress (salinity, desiccation) (for review see Kamyiama et al., 2021). The physiological role of SnRK2s from group 1 (SnRK2.1, SnRK2.4, SnRK2.4, SnRK2.9, and SnRK2.10) is relatively poorly understood. In 2012, McLoughlin et al. (2012) established a role of SnRK2.4 and SnRK2.10 in the response to salt stress. SnRK2.4 was found to stimulate primary root growth, SnRK2.10 lateral root density under salt stress, whereas SnRK2.1, SnRK2.5, and SnRK2.9 were shown to control root growth under non-stress conditions. It has also been shown recently that ABA non-activated SnRK2s phosphorylate VARICOSE (VCS), an mRNA decapping activator, and thus regulate mRNA decay under osmotic stress conditions and osmotic stress-dependent transcript accumulation (Soma et al., 2017; Kawa et al., 2020). Our recent phosphoproteomic study suggested that SnRK2.10 phosphorylates two dehydrin proteins, EARLY RESPONSIVE TO DEHYDRATION 10 and 14, in response to osmotic stress (Maszkowska et al., 2019). Moreover, SnRK2.10 conditions the plant tolerance to salinity by helping to maintain photosynthetic efficiency through the protection of the photosynthetic machinery from salinity-caused damage and diminution of ROS accumulation (Mazur et al., 2021). SnRK2.4 is also a negative regulator of root growth in the presence of cadmium ions and putatively influences iron homeostasis (Kulik et al., 2012). There is additional data indicating that the ABA non-activated SnRK2s are activated by salinity, drought, bacterial elicitors, and oxidative stress in other plant species and potentially could regulate their tolerance to those factors (Kulik et al., 2015; for review see Kulik et al., 2011 and Fàbregas et al., 2020).

Salinity induces the accumulation of ROS in plant cells, including hydrogen peroxide (H_2_O_2_), superoxide anion radical (O_2_^·-^), hydroxyl radical (OH^.^), and singlet oxygen (^1^O_2_). In response to different types of environmental stresses, ROS are mainly generated in chloroplasts, but also in mitochondria, peroxisomes, and on the outside of the cell membrane (Sewelam et al., 2014; Smirnoff and Arnaud, 2019; Liu et al., 2021). The ROS formed upon salinity have two distinct roles, both positive and negative. Those generated at the early stages of stress mostly serve as an early warning system triggering the intracellular signaling required for rapid stress response and modulation of gene expression (Allu et al., 2014; Mittler, 2017; Srivastava et al., 2021). Not all ROS have the same potential as a secondary signaling molecule. H_2_O_2_, being moderately long-lived *in vivo* (the half-life of milliseconds to seconds), can be transported and accumulated transiently in various cellular compartments and thus serve as a signal initiating distinct signaling pathways. H_2_O_2_ is involved in a cross-talk with many other signaling molecules such as other ROS and RNS (Reactive Nitrogen Species) or phytohormones, and it also modulates the activity of protein kinases (Smirnoff and Arnaud, 2019; Liu et al., 2021; Zentgraf et al., 2022). Its accumulation is controlled by scavenging enzymes, catalases and peroxidases, localized in different cellular compartments (Sewelam et al., 2016; Mittler, 2017; Zentgraf et al., 2022). When a stress is too strong the balance between ROS production and scavenging is disturbed and ROS accumulate uncontrollably causing damage to proteins, nucleic acids, and lipids, which leads to irreversible defects in cell functioning and eventually to its death (Hasanuzzaman et al., 2021; Srivastava et al., 2021). The extent of ROS accumulation and oxidative damage vary among distinct plant organs. Although an accumulation of ROS, in particular H_2_O_2_, has been observed under salinity in both roots and leaves (Szymańska et al., 2019; Mazur et al., 2021; for review see Hasanuzzaman et al., 2021), the roots suffer more damage than the rest of the plant, because they are usually the first to be exposed to salinity (Hasanuzzaman et al., 2021). The ability to cope with an excessive accumulation of salt-induced ROS is often correlated with the resistance of individual genotypes to salinity. This suggests that the type of ROS accumulated, their level and the site of the accumulation determine the overall response to salinity stress and are subject to subtle regulation (Murata et al., 2012; Zentgraf et al., 2022).

Transcription factors (TFs) are important constituents of plant signaling pathways that determine long-term (i.e., those involving changes in gene expression) responses to biotic and abiotic stimuli (Joshi et al., 2016). Large-scale transcriptomic analyses have revealed that the response to salinity is regulated in *Arabidopsis* by numerous transcription factors from distinct families, among others WRKY (Seki et al., 2002; Allu et al., 2014), which is a unique superfamily of TFs in higher plants and algae playing important roles in diverse life processes, and biotic and abiotic stress responses (Eulgem et al., 2000; Zhang and Wang, 2005; Li et al., 2020). Their functioning is regulated at the transcriptional, post-transcriptional, and post-translational levels (Giacomelli et al., 2012; Phukan et al., 2016). Expression of *WRKY* genes is controlled by TFs belonging to other families and by WRKYs. The cellular abundance, properties and activity of the WRKY TFs are modulated by ubiquitination (Yu et al., 2013), phosphorylation (Qiu et al., 2008a; Xie et al., 2010; Adachi et al., 2015), and interactions with other signaling components, including other WRKYs (by homo- and heterodimerization) (Chi et al., 2013). They can also be controlled through inter-organelle retrograde signaling; for example, the AtWRKY18-40-60 cluster is regulated by chloroplast-mediated retrograde signals (Shang et al., 2010).

Owing to their high plasticity and responsiveness to a broad range of environmental and intracellular signals, the WRKY TFs are an interesting but complex model to study the integration and differentiation of cellular signaling pathways and responses. Not all upstream factors modulating their abundance and activity have been identified, including the numerous SnRK2s. Here we focused on the poorly characterized SnRK2.10 and studied its effects on *WRKY* expression under salinity and oxidative stress. We monitored the expression levels of four *WRKY* genes, *WRKY33, WRKY40, WRKY46*, and *WRKY75*, upon salinity, H_2_O_2_ application, and oxidative stress evoked by treatment of plants with MV in wild-type and *snrk2.10* insertion mutants. Putative targets of the WRKY33, WRKY40, WRKY46 and WRKY75 TFs were predicted by a bioinformatic analyses of data collected from numerous data bases. The four WRKY TFs showed highly similar target specificity, which included numerous genes encoding stress-responsive protein kinases.

## Materials and methods

### Plant lines and growth conditions

The *Arabidopsis thaliana* lines used in the study: wild-type Col-0, *snrk2.10-1* (WiscDsLox233E9), and *snrk2.10-3* (SAIL_698_105). The line expressing SnRK2.10-GFP was kindly provided by Prof. Christa Testerink (Wageningen University).

For salinity-dependent gene expression analysis plants were grown under short-day conditions in a hydroponic culture (Araponics system) as previously described (Mazur et al., 2021). For MV-dependent gene expression analysis plants were grown in soil for three weeks.

For aseptic hydroponic cultures, seeds were sterilized by gentle shaking in 70% ethanol for 2 min, then incubated in water: bleach solution (13:1, v:v) for 20 min and washed five times with sterile water. About 100 seeds were planted to glass flasks containing 100 mL of ½ Murashige and Skoog medium supplemented with ½ Murashige and Skoog vitamin solution, 500 mg/L MES, 10 g/L sucrose, pH 5.7 and imbibed at 4°C for 5 d. Seedlings were grown for the next 10 days under constant shaking at 22°C and long-day conditions.

### Stress application

For salinity-dependent gene expression analysis and H_2_O_2_ visualization five-week-old plants were treated or not (control) with 150 mM NaCl for up to six days and whole rosettes were collected for further analysis as described (Mazur et al., 2021).

Treatment with H_2_O_2_ was performed on seedlings grown in aseptic hydroponic cultures. For gene expression analysis the seedlings were incubated with 10 mM H_2_O_2_ for 5 h, as described (Allu et al., 2014). For SnRK2.10 kinase activity analysis, the seedlings were treated with 2 mM H_2_O_2_ for up to 2 h, according to (Kulik et al., 2012), with 250 mM NaCl for 5 min, or with 50 µM MV for up to 2 h. The seedlings were collected immediately after the treatment and frozen in liquid nitrogen.

For MV-dependent gene expression analysis and H_2_O_2_ visualization, soil-grown three-week-old plants were sprayed on the abaxial and adaxial sides of leaves with 25 µM or 50 µM MV respectively, and incubated under illumination for 7 h as described (Benina et al., 2015). Only fully developed leaves were collected for further analysis.

### Immunoprecipitation and Immunocomplex kinase activity assay

The procedure was performed as described in Mazur et al. (2021). In brief, for immunoprecipitation of GFP-fused proteins, 400 µg of crude protein extract from seedlings treated with H_2_O_2_ or MV was incubated with 10 µL of GFP-Trap^®^_A (Chromotek) for 2.5 h with gentle rocking. After intensive washing, agarose beads with bound immunocomplexes were suspended in 20 mM Tris–HCl, pH 7.5 supplemented with 150 mM NaCl and 4 µg of Myelin Basic Protein (Sigma-Aldrich) per sample. To each sample, ATP supplemented with 1 μCi of [γ^32^P]ATP in kinase buffer (25 mM Tris-HCl, pH 7.5, 5 mM EGTA, 1 mM DTT, 30 mM MgCl_2_) was added to 50 μM final concentration. After 15 min of incubation at 37°C samples were mixed with Laemmli sample buffer and incubated for 3 min at 95°C with vigorous shaking. Proteins were separated on 12% SDS polyacrylamide gel and signal was detected on Medical X-ray Blue/MXBE Film (Carestream).

### Immunoblotting

Immunoblotting was performed as previously described (Mazur et al., 2021). PVDF membranes were stained with 0.2% Ponceaus S for protein loading visualization. For the detection of GFP-conjugated protein, HRP-conjugated anti-GFP antibody (Santa Cruz Biotechnology, USA) diluted 1:1000 was used according to the manufacturer’s protocol.

### RT-qPCR

Rosettes were ground to a fine powder in liquid nitrogen. RNA was extracted with Trizol (Molecular Research Center) according to the manufacturer’s instructions and treated with DNase 1 (Thermo Scientific). Reverse transcription was performed on 1 µg of RNA using the RevertAid First Strand cDNA Synthesis Kit (Thermo Scientific). The resulting cDNA was diluted tenfold with water and 1 μL of the sample was used for qPCR in a Step One Plus device (Applied Biosystems) using the GoTaq^®^ qPCR Master Mix (Promega) and specific pairs of primers (**Supplemental Table 1**). Expression levels were calculated relative to the housekeeping genes *PDF2* (*At1g13320*) and *PEX4 (At5g25760)* using the delta-delta Ct method.

### Statistical analysis

Statistical analysis was performed by One Way ANOVA followed by *post hoc* Tukey’s test (p < 0.05).

### Intracellular localization of H_2_O_2_

For detection of H_2_O_2_ localization in response to salinity plants were grown for 5 weeks in a hydroponic system (Mazur et al., 2021). For the experiment, we employed two ways of salt stress application. First, we added NaCl directly into the growing media (root application): 250 mM for 30 min or 150 mM NaCl for 3 days. The undetached leaves were stained with 50 µM BES-H_2_O_2_-Ac probe (Wako Chemicals) in 10 µM PIPES buffer, pH 6.8 for 30 min, cut from the rosette, washed 5 times for 1 min in the above buffer and observed immediately.

In the second attempt (direct treatment) the undetached leaves were firstly stained as described above and then cut from the plant and treated with 150 mM NaCl or 250 mM in 10uM PIPES buffer, pH 6.8 and immediately examined under the confocal microscope. Images were taken after 30 min of the salt treatment.

For monitoring of paraquat-triggered H_2_O_2_ accumulation in leaf cells, plants grown for 3 weeks in soil were sprayed with 50 µM MV and incubated on light for 2 to 7 h, and stained with BES-H_2_O_2_-Ac as described above.

The BES-H_2_O_2_-Ac fluorescence from leaf tissues was registered with the Nikon EZ-C1 confocal microscope using an excitation light at a wavelength of 488nm set at 1% of maximum power (20mW, Sapphire, Coherent, USA). The emission of BES-H_2_O_2_-Ac was collected with 515/30 emission filter and displayed in false green. Simultaneously the chlorophyll autofluorescence was detected by a 610 long pass filter and displayed in false magenta. The pinhole and exposure time were optimized and all settings of fluorescence detection were the same for experiments. Leaf samples have been imaged in the epidermis layer and first layer of spongy mesophyll cells using 20x oil immersion objective (Nikon, CFI Plan Fluor NA 0.75) and 60x oil immersion objective (Nikon, CFI Plan Apochromat NA 1.4) Single confocal sections and stacks were collected in Nikon EZ-C1 software. The images were digitally processed using FIJI software and the figures compiled in FigureJ plugin (NIH, Bethesda, MD, USA) (https://journals.plos.org/plosone/article?id=10.1371/journal.pone.0240280).

### Bioinformatic resources and analysis

Protein interactors of WRKY33, WRKY40, WRKY46, and WRKY75 were extracted from the following databases: BioGRID 3.5.187 (Oughtred et al., 2019), TAIR Interactome 2.0 (Berardini et al., 2015), STRING 11.0 (Szklarczyk et al., 2019), and Kihara Bioinformatics Laboratory’s resources (Ding and Kihara, 2019). Predicted target genes of WRKY33, WRKY40, WRKY46, and WRKY75 were extracted from Expresso (Aghamirzaie et al., 2017), PlantRegMap (Tian et al., 2020), Plant Cistrome Database (O’Malley et al., 2016), AthaMap (Hehl et al., 2016), and Chip-seq experimental data (Birkenbihl et al., 2017). For validation and GO term analysis, the predicted target genes were analyzed with TF2Network (Kulkarni et al., 2018). The Venny 2.1 online resource (Oliveros, 2007-2015) was used to create Venn diagrams. BioMart (Durinck et al., 2009) was used to enlist the information related to the obtained genes.

## Results

### SnRK2.10 controls the expression of several WRKY TFs under salinity

**Figure 1:**
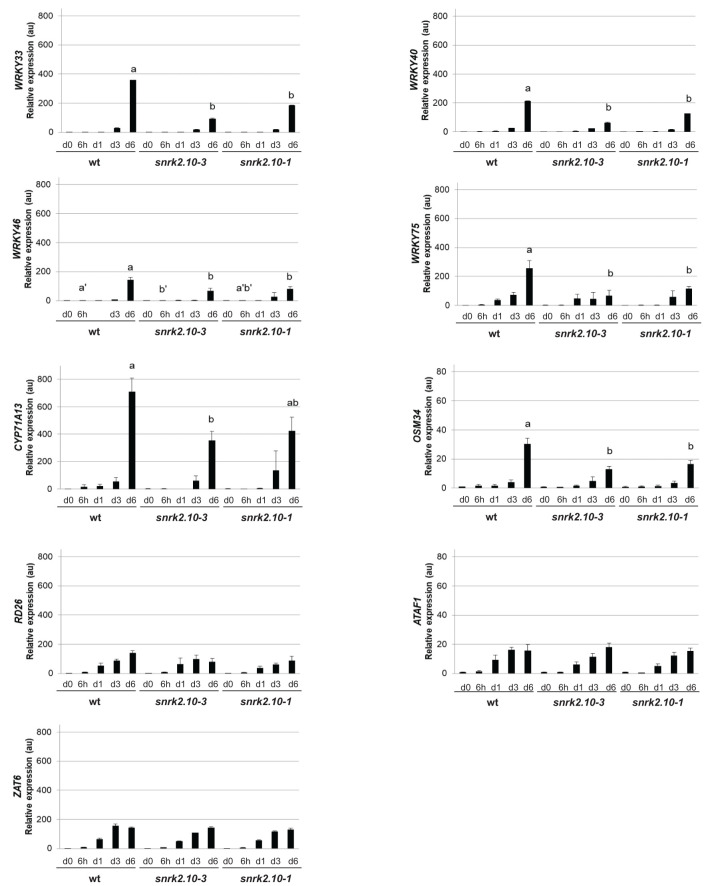
Effect of SnRK2.10 gene disruption on the expression of selected genes upon salinity. Transcript level was determined by RT-qPCR in rosettes of five-week-old plants not treated (day 0; d0) or treated with 150 mM NaCl for 6 hours (6h), 1, 3 and 6 days (d1, d3, d6), and normalized against *PEX4* (*At5g25760*) gene. The expression of each gene is shown relative to that in non-treated wild-type plants (Col-0). Mean values from three independent biological replicates, each with 8-10 plants from each line, +/- SD are shown. Statistical significance of differences between groups was determined by ANOVA and Tukey *post hoc* test. The same letters denote values ​​belonging to the same homogeneous group (p < 0.05).

During the past twenty years, an extensive transcriptomic analysis of the action of ABA-activated SnRK2s (SnRK2.2, SnRK2.3, and SnRK2.6) under osmotic stress, salinity, and ABA treatment has identified numerous genes regulated by these kinases, including many encoding transcription factors, which helped us to understand the mechanisms by which the ABA-activated SnRK2s determine plant resistance to water deficiency (Fujita et al., 2009). However, the genes regulated by the ABA non-activated SnRK2s, and particularly SnRK2.10, were characterized only partially. To begin filling this gap we compared the expression of selected genes in wt *Arabidopsis* and *snrk2.10* mutants exposed to salinity for up to six days. SnRK2.10 was found to play a major role in the induction of four genes from the WRKY family of transcription factors (**Figure 1**). Prior to salinity treatment, the basal expression of the genes in question was the same in wt plants and the mutants. Following exposure to salinity, the expression of *WRKY33, WRKY40*, and *WRKY46* was induced slightly in the wt after three days of treatment and at day six reached maximum values of approximately 360-, 210-, and 140-fold induction, respectively. The expression of *WRKY75* was induced already on day one of salinity and reached 240-fold induction on day six. Notably, in the both tested *snrk2.10* mutants the induction of these genes on the sixth day of salinity was significantly lower than in wild-type plants. Next, we analyzed the expression of *CYTOCHROME P450, FAMILY 71, SUBFAMILY A, POLYPEPTIDE 13* (*CYP71A13*), which is a direct target gene of WRKY33 and WRKY40 (Birkenbihl et al., 2012; Birkenbihl et al., 2017), and of *OSMOTIN 34* (*OSM34*), a target of WRKY33 (Zheng et al., 2006; Jiang et al., 2007). The expression of *CYP71A13* and *OSM34* increased in all plant lines, reaching about 700- and 30- fold induction, respectively, on day six of salinity, in the wild-type plants, and only ca. half of those values in the *snrk2.10* mutants. These results indicate that the expression of *WRKY33, WRKY40, WRKY46*, and *WRKY75* and of two of their targets is regulated by a SnRK2.10-dependent signaling pathway(s).

Next, we analyzed the expression of transcription factors belonging to other families. The expression of *RESPONSIVE TO DESSICATION26/ANAC072* (*RD26*) and *ARABIDOPSIS TRANSCRIPTION ACTIVATOR FACTOR 1* (*ATAF1*), both genes belonging to the *Arabidopsis* NAC Domain Containing Protein (ANAC) family and involved in the maintenance of tolerance to salinity and water regime (Wu et al., 2009; Liu et al., 2016; Ye et al., 2017), was continuously rising in the wt plants and in both *snrk2.10* mutants upon salinity. On day six the expression of *RD26* was increased nearly 150-fold in the wt and ca. 100-fold in the mutant, and of *ATAF1* over 15-fold in all three lines (**Figure 1**).

As a representative of the C2H2 zinc finger family of transcription factors we analyzed the expression of *ZINC FINGER OF ARABIDOPSIS THALIANA 6* (*ZAT6*) known to positively regulate seed germination and plant tolerance to salinity (Liu et al., 2013; Shi et al., 2014; Tang and Luo, 2018). Its expression was rising continuously upon salinity and on day six it was 150 times higher than on day zero in all three lines (**Figure 1**).

### A cross-talk between WRKY33, WRKY40, WRKY46, and WRKY75 gene targets and protein interactors

**Figure 2:**
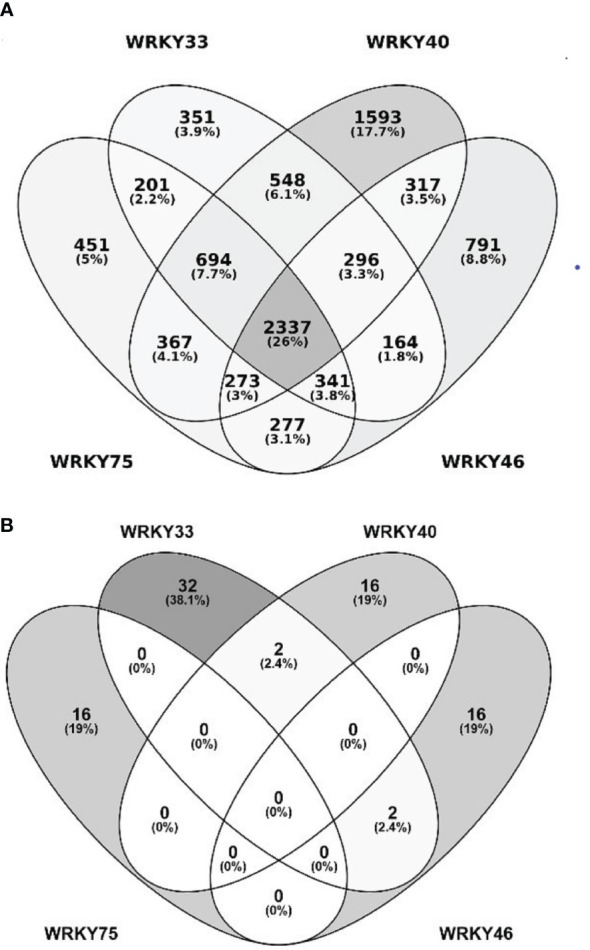
WRKY33, WRKY40, WRKY46, and WRKY75 target genes and interacting proteins. **(A)** Number of predicted target genes of WRKY33, WRKY40, WRKY46, and WRKY75. Color intensity reflects the number of genes. **(B)** Number of protein partners of WRKY33, WRKY40, WRKY46, and WRKY75.

The transcription factors from the WRKY superfamily are involved in plant responses to diverse biotic and abiotic stresses (Phukan et al., 2016; Li et al., 2020; Wani et al., 2021). They often function as homo- or heterodimers, heterooligomers, or act redundantly to each other. Owing to those features, the genes regulated by individual WRKY TFs frequently overlap, which allows specific functional groups to be discerned within the WRKY family (Li et al., 2020; Wani et al., 2021). We, therefore, conducted a comparative bioinformatic analysis of putative target genes of WRKY33, WRKY40, WRKY46, and WRKY75 extracted from five databases (see Materials and methods for details). We found 4941, 4932, 6425, and 4796 putative target genes of WRKY33, WRKY40, WRKY46, and WRKY75, respectively (**Figure 2A** and **Supplemental Data Sheet 1**). About 351, 1593, 791, and 451 of those were specific for the respective TFs, while ca. 26% (2337) genes were ranked as regulated by all four TFs. These common WRKY target genes were subjected to a functional analysis using the Gene Ontology (GO) categorization. The most significantly enriched categories included genes encoding proteins having a kinase activity, mainly protein serine-threonine kinase activity (**Figure S1**). These kinases belong to very distinct families and are involved in plant responses to multiple environmental biotic and abiotic stress factors (**Table 1**).

**Table 1:** Stress-responsive protein kinases, potential targets of WRKY33, WRKY40, WRKY46, and WRKY75.

Gene ID	Gene name	Gene description	Involvement in stress response	Ref.
MAPK				
AT1G59580 AT1G07880 AT2G01450 AT4G26070 AT4G29810 AT5G66850 AT2G30040	MPK2 MPK13 MPK17 MKK1 ATMKK2 MAPKKK5 MAPKKK17	Mitogen-activated protein kinase 2 Mitogen-activated protein kinase 13 Mitogen-activated protein kinase 17 NMAPKK MAP kinase kinase 2 Mitogen-activated protein kinase kinase kinase 5 Mitogen-activated protein kinase kinase kinase 17	Induction of SA-mediated leaf senescence, response to *Psm* response to wounding, JA, ABA and H_2_O_2_ Response to flg22 Response to salinity Defense response, response to H_2_O_2_ Defense response, response to chitin, cold and salinity Response to salinity, ABA and wounding	Ortiz-Masia et al., 2007; Sözen et al., 2020; Zhang et al., 2020 Nitta et al., 2014 Frick and Strader, 2018 Teige et al., 2004; Gao et al., 2008; Qiu et al., 2008b; Kong et al., 2012 Teige et al., 2004; Gao et al., 2008; Qiu et al., 2008b; Kong et al., 2012 Yamada et al., 2016; Sun et al., 2018; Yan et al., 2018 Choi et al., 2017; Sözen et al., 2020
SnRK				
AT3G29160 AT1G48260 AT5G35410	KIN11 CIPK17 (SnRK3.21) CIPK24 (SnRK2.11)	SNF1 Kinase Homolog 11 CBL-interacting serine/threonine-protein kinase 17 CBL-interacting serine/threonine-protein kinase 24	Response to energy deprivation, response to ABA and multiple abiotic stress factors Response to salinity, osmotic stress and ABA Response to salinity and ABA	Baena-González et al., 2007; Jossier et al., 2009; Chen and Hoehenwarter, 2015 Hrabak et al., 2003; Song et al., 2018 Hrabak et al., 2003; Yang et al., 2019; Lou et al., 2020
CDPK				
AT1G76040 AT3G57530 AT4G04700 AT4G04720	CPK29 CPK32 CPK27 CPK21	Calcium-dependent protein kinase 29 Calcium-dependent protein kinase 32 Calcium-dependent protein kinase 27 Calcium-dependent protein kinase 21	Response to auxin Response to ABA and salinity, regulation of nitrogen homeostasis Response to salinity Response to ABA, redox changes and salinity	Lee et al., 2021 Choi et al., 2005; Liu et al., 2017; Qin et al., 2020 Zhao et al., 2015 Geiger et al., 2010; Ueoka-Nakanishi et al., 2013; van Kleeff et al., 2018
RLK				
AT5G24430	CRK4	CYSTEINE-RICH RLK (RECEPTOR-LIKE PROTEIN KINASE) 4	Response to pathogens and SA	Du and Chen, 2000
AT4G23130	CRK5	cysteine-rich RLK (RECEPTOR-like protein kinase) 5	Response to *Pseudomonas syringae*, UV and ABA	Chen et al., 2003; Burdiak et al., 2015; Lu et al., 2016
AT4G23140	CRK6	cysteine-rich RLK (RECEPTOR-like protein kinase) 6	Response to *Pseudomonas syringae* and ROS	Idänheimo et al., 2014; Yeh et al., 2015
AT1G11350	SD113	G-type lectin S-receptor-like serine/threonine-protein kinase SD1-13	Defense response	Kim et al., 2009
AT1G61380	SD129	G-type lectin S-receptor-like serine/threonine-protein kinase SD1-29	Response to *Pseudomonas syringae*	Ranf et al., 2015; Luo et al., 2020
AT1G14370	BPL2	PBS1-LIKE 2	Response to *Xanthomonas campestris*	Guy et al., 2013; Wang et al., 2015
AT5G20480 AT3G21630 AT4G33430 AT5G46330 AT4G08850	EFR CERK1 BAK1 FLS2 MIK2	EF-TU RECEPTOR Chitin elicitor receptor kinase 1 BRI1-associated receptor kinase FLAGELLIN-SENSITIVE 2 MDIS1-interacting receptor like kinase 2	Defense response Response to chitin and fungal pathogens Defense response, response to mechanical stress Defense response Defense response, response to salinity	Zipfel et al., 2006; Xiang et al., 2008; Yuan et al., 2021 Miya et al., 2007; Wan et al., 2008 Chinchilla et al., 2007; Heese et al., 2007; Yasuda et al., 2017; Okamoto et al., 2021 Gómez-Gómez and Boller, 2000; Zipfel et al., 2004; Chinchilla et al., 2007; Van der Does et al., 2017; Coleman et al., 2020; Rhodes et al., 2021
AT2G23770	LYK4	LysM domain receptor-like kinase 4	Response to chitin	Wan et al., 2012; Xue et al., 2019
AT2G33580	LYK5	LysM domain receptor-like kinase 5	Response to chitin	Cao et al., 2014; Huang et al., 2020
AT1G51800	IOS1	IMPAIRED OOMYCETE SUSCEPTIBILITY 1LRR receptor-like serine/threonine-protein kinase	Response to *Hyaloperonospora arabidopsidis*, Pseudomonas syringae, chitin and BABA; modulation of ABA signaling	Hok et al., 2011; Hok et al., 2014; Yeh et al., 2016
AT1G16150	WAKL4	Wall-associated receptor kinase-like 4	Response to Na^+^ (by NaCl treatment), K^+^, Cu^2+^, Ni^2+^, and Zn^2+^	Hou et al., 2005
AT1G16160	WAKL5	Wall-associated receptor kinase-like 5	Defense response mediated by SA, response to wounding	Verica et al., 2003
AT2G19190	SIRK	Senescence-induced receptor-like serine/threonine-protein kinase	Response to flg22, *Pseudomonas syringae*, leaf senescence	Robatzek and Somssich, 2002; He et al., 2006
AT3G09830	PCRK1	Serine/threonine-protein kinase PCRK1	Response to *Pseudomonas syringae*	Sreekanta et al., 2015; Kong et al., 2016
AT5G01550	LECRKA4.2	lectin receptor kinase a4.1	Response to ABA	Xin et al., 2008
AT5G60300	LECRK19	L-type lectin-domain containing receptor kinase I.9	Response to extracellular ATP, *Botrytis cinerea* and *Rhizoctonia solani*	Tripathi et al., 2018; Wang et al., 2018; Kumar et al., 2020
AT4G04960	LECRK71	L-type lectin-domain containing receptor kinase VII.1	Response to *Pseudomonas syringae*	Yekondi et al., 2018
AT4G28490	RLK5	Receptor-like protein kinase 5	Response to *Pseudomonas syringae*,	Wang et al., 2017;
S6K				
AT3G08730	ATPK1	ARABIDOPSIS THALIANA PROTEIN-SERINE KINASE 1	Response to osmotic stress and other factors regulating RAPTOR-dependent signaling pathways	Mahfouz et al., 2006; Obomighie et al., 2021
AT3G08720	ATPK2	Serine/threonine-protein kinase AtPK2/AtPK19	Response factors regulating RAPTOR-dependent signaling pathways	Mahfouz et al., 2006
Others				
AT1G68830 AT1G71697 AT3G25250 AT5G63770	STN7 CEK1 OXI1 DGK2	Serine/threonine-protein kinase STN7, chloroplastic CHOLINE/ETHANOLAMINE KINASE 1 Serine/threonine-protein kinase OXI1 Diacylglycerol kinase 2	Response to high and fluctuating light, response salinity, redox changes and oxidative stress Response to salinity, ER stress Response to ROS, Cu^2+^ and high light, defense response Response to freezing	Bellafiore et al., 2005; Chen and Hoehenwarter, 2015 Tasseva et al., 2004; Lin et al., 2019 Rentel et al., 2004; Petersen et al., 2009; Smeets et al., 2013; Shumbe et al., 2016 Tan et al., 2018

In contrast to the highly overlapping gene target sets, the protein partners of WRKY33, WRKY40, WRKY46, and WRKY75 showed high specificity towards particular transcription factors (**Figure 2B**). Thirty-two proteins have previously been identified as interacting specifically with WRKY33, and 16 each with WRKY40, WRKY46, and WRKY75. In addition, four proteins interacted with two of the TFs. Among specific interactors, protein kinases involved in stress response and transcription factors were particularly abundant (**Supplemental Data Sheet 2**).

### SnRK2.10 is activated under oxidative stress and controls the expression of several WRKY TFs

**Figure 3:**
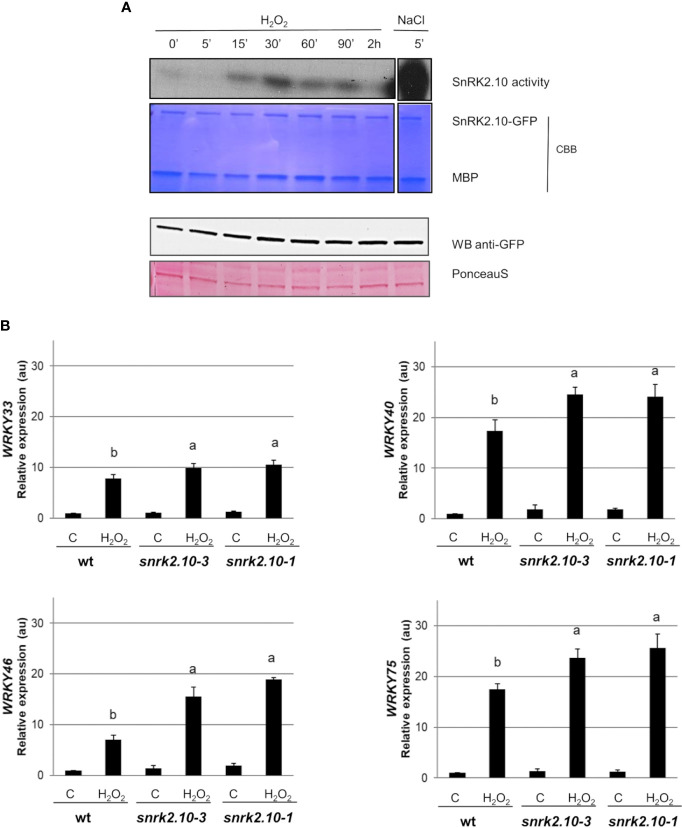
Involvement of SnRK2.10 in plant response to H_2_O_2_. **(A)** SnRK2.10 activity. Seedlings of transgenic *Arabidopsis* line expressing SnRK2.10-GFP were grown in hydroponic culture for ten days and exposed or not to 2 mM H_2_O_2_ for up to 2 hours. Kinase activity was determined by in-gel kinase activity assay with γ-[^32^P] ATP and MBP (myelin basic protein) as substrates. SnRK2.10-GFP protein was quantified by western blotting with anti-GFP antibodies. A representative result of three independent repeats is shown. CBB – Coomassie Brilliant Blue. **(B)** Expression of selected genes. Transcript level was monitored by RT-qPCR in ten-day-old seedlings not treated (C) or treated with 10 mM H_2_O_2_ for 5 hours (H_2_O_2_), and normalized against *PEX4* (*At5g25760*) gene. The expression of each gene is shown relative to that in non-treated wild-type plants (Col-0). Mean values from three independent biological replicates, each with 8-10 plants from each line, +/- SD are shown. Statistical significance of differences between groups was determined by ANOVA and Tukey *post hoc* test. The same letters denote values ​​belonging to the same homogeneous group (p < 0.05).

The ROS accumulated during the early response to biotic and abiotic stress factors function as secondary messengers and play a substantial role in triggering cellular signaling. To investigate the role of SnRK2.10 in the ROS-mediated signaling we focused on H_2_O_2_ because of its relatively long life and high mobility (Smirnoff and Arnaud, 2019; Liu et al., 2021). To investigate whether H_2_O_2_ can activate SnRK2.10 in the absence of salt stress, transgenic seedlings expressing SnRK2.10-GFP were treated with 2 mM hydrogen peroxide for up to two hours (**Figure 3A**). The SnRK2.10 activity was detected after fifteen minutes of the exposure, reached a maximum after 30 minutes, and returned to the control level after two hours. The activity triggered by H_2_O_2_ treatment was lower than that observed after 5 min of salinity. The level of the SnRK2.10-GFP protein did not change during the treatment.

According to Allu et al. (2014) *WRKY33, WRKY40*, and *WRKY75* are upregulated in plants in response to ROS/H_2_O_2_ treatment. To determine whether SnRK2.10 contributes to this induction, ten-day-old seedlings of the wt and the *snrk2.10* mutants were treated with 10 mM H_2_O_2_ as previously described (Allu et al., 2014). In all three lines the expression of the *WRKY33, WRKY40, WRKY46*, and *WRKY75* genes was significantly increased compared to control levels (**Figure 3B**). Although, the induction of *WRKYs* expression in H_2_O_2_ treated samples was lower than after salinity stress (**Figure 1**). In wt plants the increase was, respectively, 8-, 17-, 7-, and 17-fold, while in the *snrk2.10* mutants it was significantly higher: 10-, 25-, 16-, and 25-fold, respectively (**Figure 3B**). The activation of SnRK2.10 by salinity (**Figure 1**) had an opposite effect on WRKY expression to the activation in response to H_2_O_2_ (**Figure 3**), suggesting the existence of two distinct signaling pathways involving SnRK2.10 and triggering different stress-specific transcriptional responses.

### SnRK2.10 is not activated by methyl viologen-generated ROS

**Figure 4:**
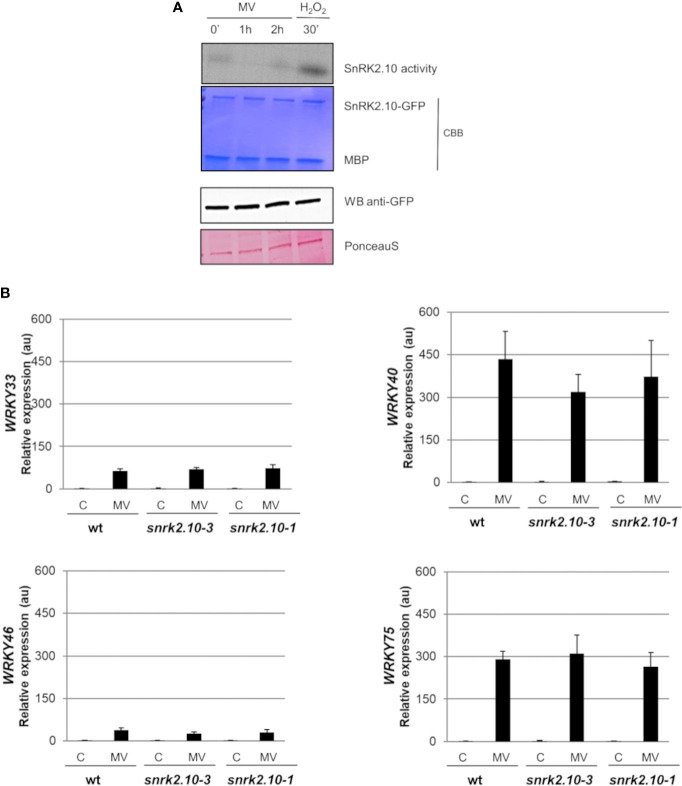
Involvement of SnRK2.10 in plant response to methyl viologen. **(A)** SnRK2.10 activity. Seedlings of transgenic *Arabidopsis* line expressing SnRK2.10-GFP were grown in hydroponic culture for ten days and exposed or not to 50 µM MV for up to 2 hours. Kinase activity was determined by in-gel kinase activity assay with γ-[^32^P] ATP and MBP (myelin basic protein) as substrates. SnRK2.10-GFP protein was quantified by western blotting with anti-GFP antibodies. A representative result of three independent repeats is shown. CBB – Coomassie Brilliant Blue **(B)** Expression of selected genes. Transcript level was monitored by RT-qPCR in rosettes of three-week-old plants not treated (C) or treated with 10 µM MV for up to 2 hours (MV) and normalized against *PEX4* (*At5g25760*) gene. The expression of each gene is shown relative to that in non-treated wild-type plants (Col-0). Mean values from three independent biological replicates, each with 8-10 plants from each line, +/- SD are shown. Statistical significance of differences between groups was determined by ANOVA and Tukey *post hoc* test (p < 0.05).

**Figure 5:**
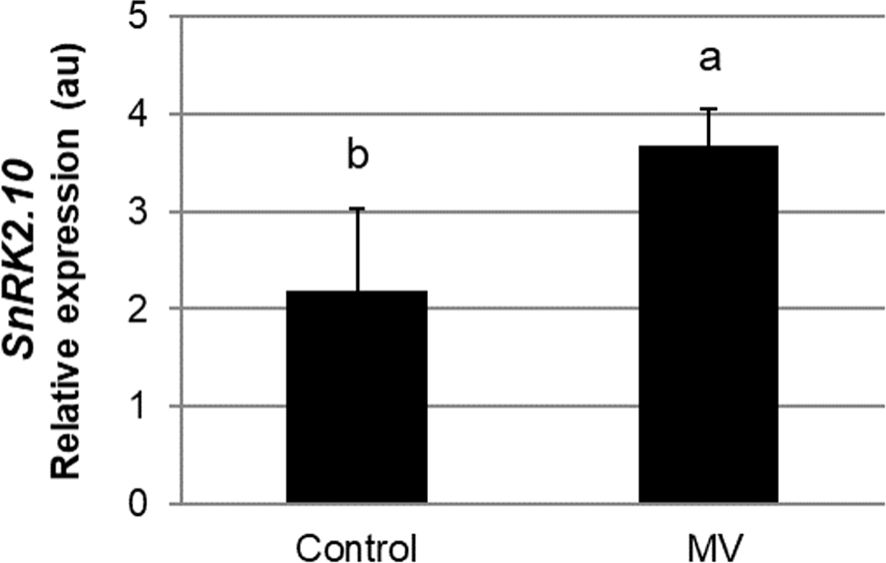
Effect of methyl viologen on *SnRK2.10* gene expression. Transcript level was monitored by RT-qPCR in rosettes of three-weeks-old plants not treated (Control) or treated with 10 µM MV for up to 2 hours (MV) and normalized against *PEX4* (*At5g25760*) gene. Mean values from three independent biological replicates, each with 8-10 plants from each line, +/- SD are shown. Statistical significance of differences between groups was determined by ANOVA and Tukey *post hoc* test (p < 0.05).

Leaf chloroplasts are very sensitive to different environmental factors, and thus can rapidly perceive and transfer the stress signal to other cellular compartments e.g., by generation of stress-specific ROS (Liu et al., 2021; Zentgraf et al., 2022). A chloroplast-specific ROS formation can be triggered by the herbicide methyl viologen (MV; *N,-N’*-dimethyl 4, -4’-bipyrydinium dichloride, also known as paraquat). Methyl viologen has been in use in field agriculture at high concentrations for ca. 60 years (Baltazar et al., 2013). In experimental plant biology, it is commonly used in low concentrations (in the nM to µM range) to study ROS signaling and oxidative stress tolerance (for review see Nazish et al., 2022). In the chloroplasts, in the presence of light, MV competes with ferredoxin for electrons on the acceptor side of photosystem I (PSI) to produce monocationic MV radical (MV˙^+^) which reacts rapidly with oxygen to form superoxide radical (O_2_˙^-^) (Hassan, 1984; Dodge, 1989; Fuerst and Norman, 1991; Hartel et al., 1992). The highly reactive O_2_^·-^ further generates other ROS, like H_2_O_2_ or OH˙, which may play signaling functions and damage the cell (Babbs et al., 1989). To determine whether SnRK2.10 can be activated by ROS generated in chloroplasts, we treated seedlings expressing SnRK2.10-GFP with 10 µM MV and analyzed the kinase activity by an in-gel assay. Only traces of SnRK2.10 activity were detected after up to 2 h of the treatment vis-a-vis a very strong signal observed following application of H_2_O_2_ (**Figure 4A**). Nevertheless, the MV treatment caused a nearly two-fold induction of the *SnRK2.10* gene, suggesting a hitherto unknown role of this kinase in the plant response to paraquat (**Figure 5**). Despite the lack of a substantial SnRK2.10 activation, the *WRKY33, WRKY40, WRKY46*, and *WRKY75* genes were induced by MV in wild-type *Arabidopsis* leaves approximately 60-, 420-, 38- and 240-fold, respectively, and to a similar extent also in the *snrk2.10* mutants (**Figure 4B**). These results indicate that SnRK2.10 does not regulate their expression in the conditions studied.

### H_2_O_2_ accumulates in leaves in a site-specific manner under salinity and MV treatments

Generation of different types of ROS, and particularly H_2_O_2_, has been widely observed in plants challenged with salinity (Mittler, 2017; Srivastava et al., 2021). These observations are performed very frequently by the histochemical staining of leaves with 3,3’-diaminobenzidine (DAB) or by measuring the hydrogen peroxide concentration in the homogenized biological materials using diverse biochemical methods (Ben Rejeb et al., 2015; Nguyen et al., 2017; Mazur et al., 2021). However, to fulfill their specific signaling role salinity-triggered ROS production must occur in specific cellular compartments. To analyze the NaCl-specific intercellular localization of H_2_O_2_, we treated *Arabidopsis* plants grown hydroponically for five weeks with 150 mM NaCl for 3 days or 250 mM NaCl for 30 min, or we not treated them (control). We then stained the leaves with an H_2_O_2_-specific fluorescent probe BES-H_2_O_2_-Ac. This fluorescent probe was previously used for the observation of dynamic hydrogen peroxide localization in plant leaves (Zhuang et al., 2021; Shi et al., 2022) and roots (Tsukagoshi et al., 2010). In our control experimental conditions, fluorescence was observed in the cytoplasm and nucleus but not in chloroplasts (**Figures 6I** and **6II, A–D**). This indicates that hydrogen peroxide is present mainly in the first two cellular compartments. After 30 min of salinity, NaCl applied to roots, the fluorescence signal was more pronounced and started to disperse more widely in the cytoplasm (**Figures 6I** and **6II, E–H**). In leaves of hydroponically grown plants treated with 150 mM NaCl for 3 days, H_2_O_2_ accumulation was widely dispersed in the cytoplasm, present in the nucleus and in many, but still not all, chloroplasts (**Figures 6I** and **6II, I–L**). It should be noted that, in leaves directly immersed in 150 or 250 mM NaCl for 30 min fluorescence was much stronger and the pattern was different. Hydrogen peroxide was present in the cytoplasm, nucleus, chloroplasts, and in numerous intensive cytoplasm-localized spots, cytoplasmic strands, and bubble-shaped structures (**Figures 6I** and **6II, M–T** and **Figure S2**). This suggests that in leaves, the NaCl-dependent accumulation site of H_2_O_2_ strongly depends on the stress duration, intensity and way of salt application, NaCl applied indirectly into the roots or directly into leave tissue. This potentially may have an impact on downstream signaling events.

**Figure 6I:**
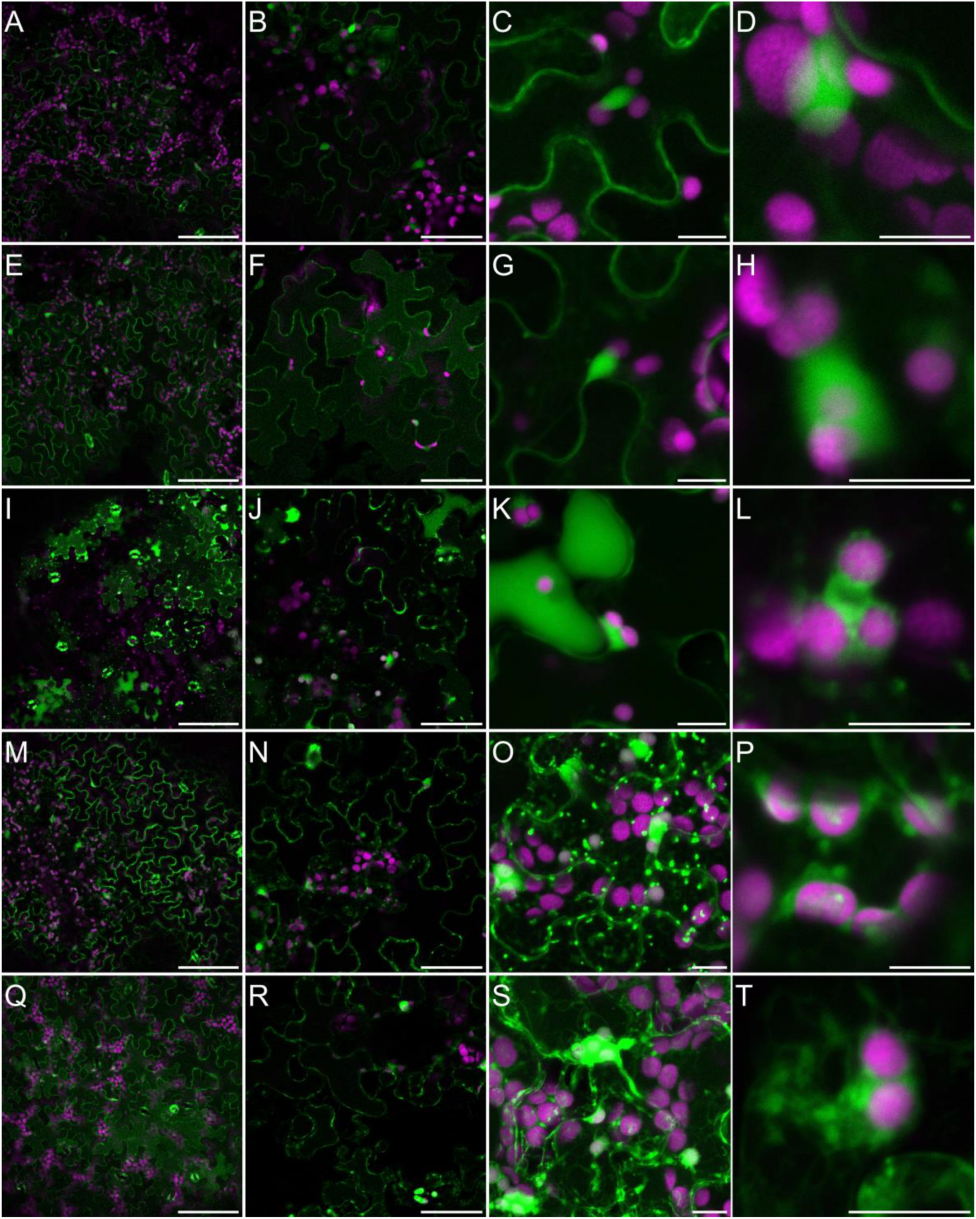
Localization of H_2_O_2_ accumulation in response to salinity. Staining of Arabidopsis leaves with an H_2_O_2_-specific BES- H_2_O_2_-Ac probe for intracellular hydrogen peroxide detection was carried out as described in Materials and Methods. Detection of BES- H_2_O_2_-Ac fluorescence is shown in false green and auto-fluorescence of chlorophyll is depicted in false magenta. The panel consists of images of leaves from control conditions **(A–D)**; 30 min of 250 mM NaCl applied to roots **(E–H)**; 3 days of 150 mM NaCl applied to roots **(I–L)**; 30 min of 150 mM NaCl applied directly to leaves **(M–P)**; 30 min of 250 mM NaCl applied directly to leaves **(Q–T)**. Scale bars: 100 μm in the first two columns and 10 μm in the last three columns. Images were made as a single scan in the first two columns, in the remaining columns merged projections from 14-25 individual optical slices are presented. Six to eight leaves from different plants and from independent biological treatments were observed for each treatment. Images from multiple optical sections were collected from each leaf. The panel shows representative images.

It has been previously shown that in leaves exposed to MV, different ROS are accumulated, among them hydrogen peroxide (Bulgakov et al., 2012; Cui et al., 2019; Wang et al., 2019). Although, the sites of its accumulation are not well documented. Thus, for monitoring of paraquat-triggered H_2_O_2_ accumulation in leaf cells, plants grown in soil were sprayed with 50 µM MV and incubated on light for 2 to 7 h, and stained with BES-H_2_O_2_-Ac. The H_2_O_2_ accumulation was observed mainly in the cytoplasm and nucleus but not in chloroplasts in both, control and MV-treated leaves (**Figure 6II**). This indicates that in response to MV H_2_O_2_ accumulates in leaf cells in the same cellular compartments/organelles as during normal growth, but must probably to higher concentrations. Alternatively, hydrogen peroxide accumulation in some cellular spaces/organelles may be very weak and beyond our detection range, since Ugalde et al. (2021) reported MV-dependent H_2_O_2_ formation in chloroplasts. This problem needs further detailed investigation and application of more sophisticated methods.

**Figure 6II:**
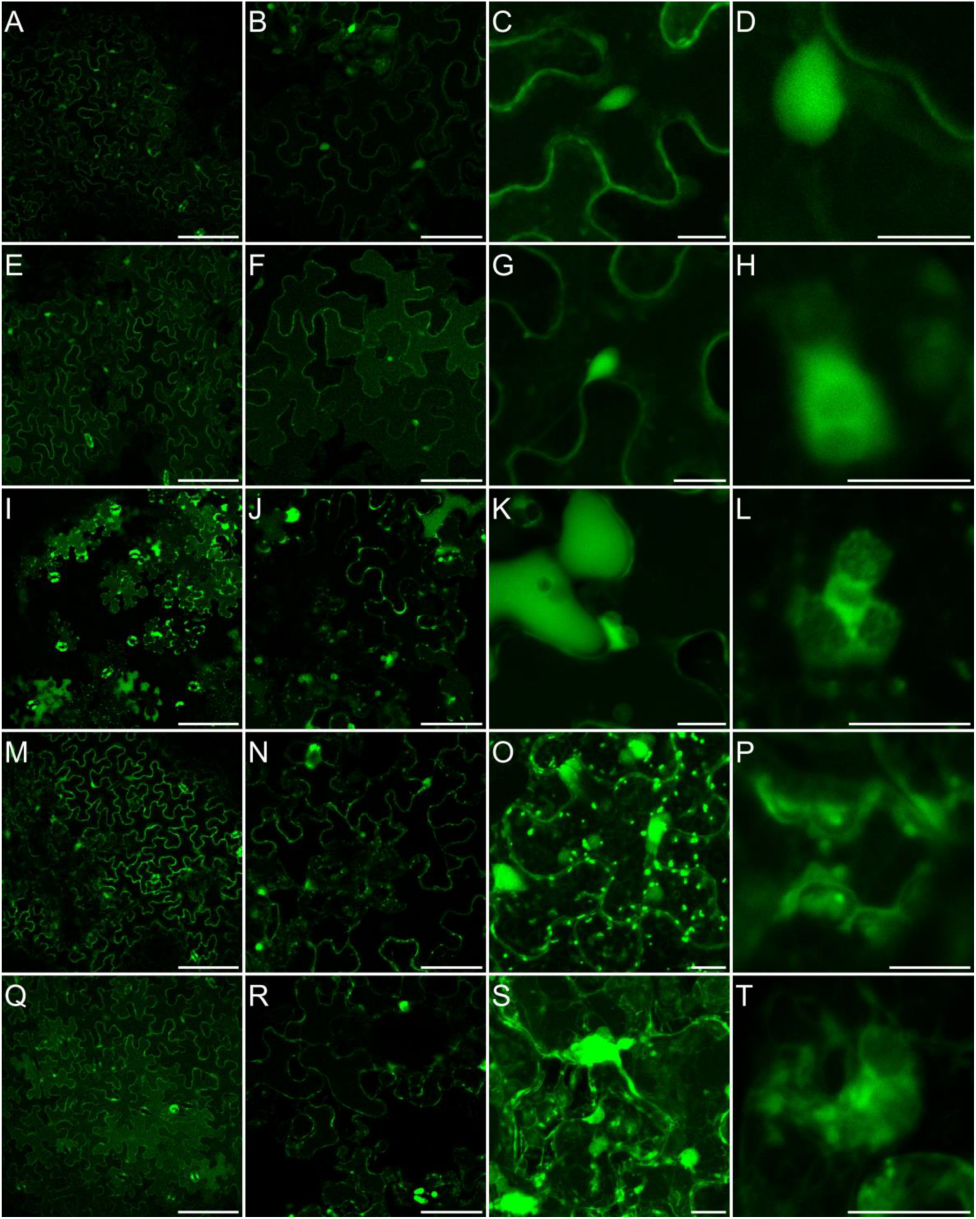
Localization of H_2_O_2_ accumulation in response to salinity. Staining of Arabidopsis leaves with an H_2_O_2_ -specific BES- H_2_O_2_-Ac probe for intracellular hydrogen peroxide detection was carried out as described in Materials and Methods. Detection of BES- H_2_O_2_ -Ac fluorescence is shown in false green. All figure captions correspond to the description of **Figure 6I**.

## Discussion

Monitoring the activation of stress-responsive kinases upon different environmental stimuli and/or the induction of their expression is the first step toward identifying their involvement in the regulation of plant responses to particular conditions. In most cases, a signal must be perceived by specific receptors to initiate a highly structured sequence of down-stream events in which the information is transduced through a branched network of intracellular pathways leading to the final response. The transfer of information about a stress is a non-linear and extremely complex process resembling an extensive root system in structure (Sewelam et al., 2016). One of the key signaling second messengers in the cell are ROS, particularly hydrogen peroxide. Several H_2_O_2_-induced protein kinases have been identified, such as MITOGEN-ACTIVATED PROTEIN KINASE 6, CALCIUM-DEPENDENT PROTEIN KINASE 5 and OXIDATIVE SIGNAL-INDUCIBLE 1 (OXI1) (Rentel et al., 2004; Wang et al., 2010; Dubiella et al., 2013). Furthermore, H_2_O_2_-dependent oxidation of specific thiol groups in type 2C protein phosphatase HYPERSENSITIVE TO ABA 1 (HAB1) inhibits its catalytic activity and the ability to interact with SnRK2.6, an ABA-activated SnRK2. This, in turn, allows auto-phosphorylation and auto-activation of SnRK2.6 and phosphorylation of its downstream targets located mainly in guard cells (Sridharamurthy et al., 2014). In our laboratory, *Nicotiana tabacum* OSMOTIC STRESS-ACTIVATED PROTEIN KINASE (NtOSAK) has been identified as a hydrogen peroxide-activated kinase, activated also by cadmium ions in an H_2_O_2_-dependent manner (Kulik et al., 2012). NtOSAK belongs to ABA non-activated SnRK2s and is closely related to SnRK2.4 and SnRK2.10 from *Arabidopsis* (Kulik et al., 2011). In this study, we showed that SnRK2.10, like NtOSAK, is activated by hydrogen peroxide. Moreover, the kinase alters the expression of four H_2_O_2_-responsive *WRKY* genes. The phosphorylation of SnRK2.10 is an element of early response to salinity and H_2_O_2_ which has been indicated by several studies including a large-scale phosphoproteomic study (Chen and Hoehenwarter, 2015). The question of whether SnRK2.10 is activated during salinity in an H_2_O_2_–dependent manner remains open for further investigation.

An opposite regulation of WRKY TFs expression by SnRK2.10 under salinity and in response to H_2_O_2_ suggests the existence of two distinct signaling pathways involving SnRK2.10 and triggering different stress-specific transcriptional responses. A differentiation of the signaling in response to H_2_O_2_ vs. prolonged salinity has already been proposed (Allu et al., 2014). To understand the specificity and identify other components of the two putative signaling pathways, further study is required, e.g., the identification of respective SnRK2.10 partners and targets under salinity and H_2_O_2_ stresses. Under salinity, VARICOSE (VCS) has been identified as an ABA-non-activated SnRK2s target in *Arabidopsis* (Soma et al., 2017; Kawa et al., 2020). VCS is a scaffold protein for DCP1 and DCP2 proteins which together form a complex that catalyzes the decapping of 5’mRNA, which is followed by the degradation of mRNA by 5′–>3′ exoribonuclease XRN4 (Sorenson et al., 2018). Within VARICOSE, multiple SnRK2.10-related phosphorylation residues have been identified. Although to date, the consequences of VCS phosphorylation remain controversial (Soma et al., 2017; Kawa et al., 2020) and we cannot undoubtedly state whether SnRK2 protein kinases enhance or inhibit 5′ mRNA decay *via* phosphorylation of VCS. Mutant plants with disrupted expression of *snrk2.4, snrk2.10, vcs* or *xrn4* show similar disturbances in the growth of the main root and lateral roots, respectively, under saline conditions (Kawa et al., 2020). It has been shown that transcripts of *WRKY33, WRKY40*, and *WRKY46* are enhanced in *vcs* and *xrn4* mutants (Basbouss-Serhal et al., 2017; Sorenson et al., 2018; Nagarajan et al., 2019; Carpentier et al., 2020). The effect of VCS-XRN4 5’mRNA decay module on WRKYs expression seems to be complex and needs further investigation, also under oxidative stress conditions. The post-transcriptional regulation mediated by the ‘subclass I SnRK2s–VARICOSE’ signaling module represents one of mechanisms of gene expression control under osmotic stress (Soma et al., 2017) and we cannot exclude other pathways by which SnRK2.10 controls *WRKY33, WRKY40, WRKY46, and WRKY75* transcription under salinity and oxidative stress. For instance, phosphoproteomic study of Maszkowska et al. (2019) revealed several putative proteins involved in mRNA metabolism phosphorylated by SnRK2.10 in roots under salinity. The schematic putative functioning of SnRK2.10-depepndent regulation of *WRKYs* expression in response to salinity has been presented on **Figure S3**.

Chloroplasts play a key role in plant functioning as the site of photosynthesis. It should be noted that they are also very sensitive sensors of environmental stresses, including salinity, and the important source of ROS generated in plants under stress (for review see Smirnoff and Arnaud, 2019; Liu et al., 2021; Zentgraf et al., 2022). Our present results show that the ROS formed upon induction of an MV-dependent oxidative burst do not induce the activity of SnRK2.10. This is perhaps unsurprising since SnRK2.10 has never been observed in chloroplasts, although its abundance in the cellular structures linking physically and functionally chloroplasts and the nucleus remains to be determined. Recently, it has been demonstrated that ROS generated locally in intact *Arabidopsis* chloroplasts by methyl viologen treatment cause dynamic changes in H_2_O_2_ accumulation in the cytosol and in mitochondria (Ugalde et al., 2021). A simple diffusion of H_2_O_2_ across cellular membranes is strongly limited, and its efficient transport occurs only through aquaporins, which are present in the plasma membrane, tonoplast, and most likely in chloroplast membranes allowing for retrograde signaling involving H_2_O_2_ moving *via* cytosol (for review see Mullineaux et al., 2019; Smirnoff and Arnaud, 2019). A second route of H_2_O_2_ transportation outside the chloroplasts is through so-called plastid-nuclear complexes and stromules (stroma-filled tubular plastid extensions) that link chloroplasts physically with the nucleus and play a pivotal role in retrograde signaling (Mullineaux et al., 2019). Salinity, drought, and ABA are among the agents inducing stromule formation (Gray et al., 2012). According to Zentgraf et al. (2022), H_2_O_2_ signaling in plants involves not only simple accumulation of the molecule but also a modulation of the ratio of H_2_O_2_ concentrations between different compartments. For instance, it has been shown that H_2_O_2_ produced in chloroplasts or in peroxisomes induces two types of transcriptomic responses: one independent of the subcellular site of H_2_O_2_ production and another that is organelle-specific (Sewelam et al., 2014). Therefore, it seems plausible that the induction of SnRK2.10-dependent signaling pathways requires not only an overall ROS accumulation in the cell but also an appropriate ROS ratio between compartments and/or specific site of ROS accumulation. Our results clearly showed that the ROS accumulation following paraquat treatment does not affect SnRK2.10-signaling in *Arabidopsis*, although the moderate induction of *SnRK2.10* expression in these conditions suggests its putative role in the plant response to the herbicide.

Identifying and establishing the roles of salt-responsive genes is key for understanding the mechanisms of the plant response to salinity as well as the molecular basis of their resistance. Transcription factors deserve special attention here because they often regulate a broad spectrum of responses to abiotic and biotic stresses. Proteins containing the WRKY domain comprise one of the largest families of transcription factors in plants and modulate numerous processes including senescence, seed development, dormancy, and germination, and diverse biotic and abiotic stress responses (Phukan et al., 2016; Birkenbihl et al., 2018).

It has been shown that *WRKY33* is induced by chloroplast-derived hydrogen peroxide in *Arabidopsis* plants overexpressing glycolate oxidase and challenged with high light conditions (Schmidt et al., 2020). On the other hand, *WRKY33* suppression leads to enhanced H_2_O_2_ accumulation (Sun et al., 2020). Jiang and Deyholos (2009) reported that *WRKY33* expression was induced under salinity and the *wrky33* null mutant showed only a moderately increased NaCl sensitivity when primary root length and ion leakage were monitored. Besides, several research groups have shown that WRKY33 strongly affects plant resistance to *Botrytis cinerea* through negative regulation of ABA biosynthesis and signaling, and by inducing synthesis of camalexin (Birkenbihl et al., 2012; Liu et al., 2015; Sham et al., 2017; Zhou et al., 2020). Further, WRKY33 together with SALT TOLERANCE ZINC FINGER (STZ, ZAT10) and ARABIDOPSIS TOXICOS EN LEVADURA 31 (ATL31) take part in a common transcriptional regulatory network inducing hypocotyl elongation downstream of the auxin perception module (Rigal et al., 2021).

Regarding WRKY40, recent research points to its role in responses to ABA, drought, and pathogens. It has been shown that the *WRKY40* gene is induced by drought and salinity, and a *wrky40* insertion mutant displays an ABA-hypersensitive phenotype in seed germination, green cotyledon formation, and primary root elongation tests (Chen et al., 2010; Rasheed et al., 2016; Ahmad et al., 2019; Wang et al., 2021; Gigli-Bisceglia et al., 2022). In cooperation with WRKY18 and WRKY60, WRKY40 modulates the plants response and susceptibility to the hemibiotrophic bacterial pathogen *Pseudomonas syringae*, and the necrotrophic fungal pathogen *Botrytis cinerea* (Xu et al., 2006; Birkenbihl et al., 2017; Abeysinghe et al., 2018).

*WRKY46* has been reported to mediate leaf senescence and undergo induction in *Arabidopsis* plants under various stresses, e.g., drought, salinity, H_2_O_2,_ and salicylic acid treatments; it confers resistance to drought and salinity by contributing to an inhibition of osmotic/salt stress-dependent formation of lateral roots *via* regulation of ABA signaling and auxin homeostasis (Ding et al., 2015; Ding et al., 2015; Chen et al., 2017; Zhang et al., 2021). The phenotypes exhibited by the *wrky46* and *snrk2.10* insertion mutant plants challenged by salinity are similar to some extent. It has been shown that *wrky46* seedlings achieve smaller increments of dry mass accumulation and exhibit higher salt sensitivity than wild-type plants, whereas WRKY46 overexpressing lines are more resistant to salinity. Seedlings of *wrky46* (Ding et al., 2014) and adult *snrk2.10* plants exposed to salinity (Mazur et al., 2021) accumulate more ROS in the leaves, suggesting that the two respective proteins may function in the regulation of cellular redox homeostasis. And finally, WRKY46 and SnRK2.10 are both engaged in lateral root formation under osmotic and salt stress (McLoughlin et al., 2012; Ding et al., 2015). Thus, there are ample indicators of a functional similarity of the SnRK2.10 and WRKY46 signaling pathways during the response to salinity or even their partial overlapping, the confirmation of which, however, requires additional research.

The WRKY75 transcription factor takes part in the signaling pathways of diverse plant hormones and acts as a multilink of the response to abiotic and biotic stressors, e.g., phosphorus starvation (Devaiah et al., 2007; Rishmawi et al., 2014), *Sclerotinia sclerotiorum* infection (Chen et al., 2013), and treatment with flagellin (Birkenbihl et al., 2018). It has been also shown that WRKY75 is induced after 24 h of salinity, plays the role of a genuine regulator of the ER-stress cellular responses, and its overexpression confers plant resistance to salt stress (Hossain et al., 2016). Besides its role in stress response, WRKY75 also participates in the regulation of plant development. In particular, it plays a role in the formation of root architecture (Devaiah et al., 2007), promotes flowering *via* gibberellin-dependent pathways (Zhang et al., 2018), mediates ABA-dependent seed germination, and senescence, where a tripartite amplification loop involving WRKY75, salicylic acid, and ROS has been reported (Guo et al., 2017; Zhang et al., 2022).

Recent studies indicate that molecular dynamics, specific homo- and heterodimerizations, as well as modular flexibility and posttranslational modifications, determine the functional specificity of many TFs engaged in environmental adaptation (Golldack et al., 2011). Among the most intensively studied TFs are those belonging to the WRKY family, notably for their propensity to function as hubs in complex protein-protein networks. Numerous WRKY hubs have been identified in biotic and abiotic stress responses, including WRKY18, WRKY33, WRKY40, WRKY46, WRKY51, WRKY53, WRKY60, and WRKY70 (Friedel et al., 2012; Chi et al., 2013; Choura et al., 2015; Birkenbihl et al., 2017). There is a significant overlap between the sets of various WRKY target genes. One such gene is *CYP71A13* encoding camalexin biosynthesis enzyme, and it is induced by NaCl treatment (Xu et al., 2008). However, the role of camalexin in the response to salinity remains unknown. The *CYP71A13* promoter is a direct target of WRKY33, WRKY40, and WRKY18, and the former two also bind to the *WRKY75* promoter in response to salinity or biotic stimuli (Birkenbihl et al., 2012; Birkenbihl et al., 2017). WRKY40 is a central node in abiotic stress response regulation in shoots, forming the regulatory network with other TFs, including WRKY33 and WRKY46. WRKY46 is a hub molecule in plant roots challenged with abiotic stress (Friedel et al., 2012). Furthermore, WRKY40 and WRKY46 act as hubs in the plant - pathogen interaction system (Biniaz et al., 2022). *OSM34* analyzed by us in this report is also controlled by WRKY TFs, e.g., WRKY33 (Zheng et al., 2006; Jiang et al., 2007). We found that under salinity the expression of the *WRKY33, WRKY40, WRKY75*, *CYP71A13*, and *OSM34* genes was reduced in *snrk2.10* mutants compared to wt plants. This suggests that SnRK2.10 affects the expression of *CYP71A13, OSM34*, and *WRKY75* by influencing their upstream regulators WRKY33 and WRKY40. Interestingly, *WRKY33, WRKY40, WRKY75*, and *CYP71A13* all belong to clusters of genes upregulated in *Arabidopsis* rosettes by H_2_O_2_ treatment and during developmental- and NaCl-induced senescence (Allu et al., 2014). This is in agreement with the observation that high and prolonged salinity can induce genes involved in programmed cell death and senescence initiation (Golldack et al., 2011). Currently, it is considered that the function of SnRK2.10 is dedicated to the response to osmotic/salt and to some extent to Cd^2+^ -induced stress (Kulik et al., 2012; McLoughlin et al., 2012; Julkowska et al., 2015; Maszkowska et al., 2019; Szymańska et al., 2019). The present results suggest that SnRK2.10, and possibly also other ABA non-activated SnRK2s, could play a more general role in the regulation of the responses to abiotic and biotic stimuli, nutrients imbalance, induction of senescence, and other developmental events by influencing the hubs of transcription factor networks. Our analysis of putative WRKY33/40/46/75 targets revealed a high proportion of genes regulated by several of those TFs and coding for proteins involved in global stress responses.

In conclusion, we have shown that NaCl-activated SnRK2.10 kinase signaling is involved in the induction of four WRKY TFs, *WRKY33, WRKY40, WRKY46*, and *WRKY75*, in *Arabidopsis* leaves, whereas H_2_O_2_-induced activation of the kinase attenuates their expression. The activation of SnRK2.10 and the following transcriptional responses do not depend on the ROS accumulation *per se*, but rather are fine-tuned depending on the source of ROS and their intracellular distribution in different compartments, and the co-occurrence with other stress factors. This indicates a previously unanticipated plasticity and variable specificity of the SnRK2.10-dependent signaling pathways, most likely achieved through interactions with other, so far very poorly understood, components and regulators. The regulation of the hub WRKY transcription factors by SnRK2.10 indicates its pivotal role in the response to abiotic stress. Analyzed by us WRKY TFs regulate a large number of stress-related protein kinases, which suggests that SnRK2.10 could modulate the pleiotropic cellular responses to salinity and ROS, including both common and stress-specific responses. Its involvement in the response to other stress factors (e.g., nutrient imbalance, pathogens) and in the regulation of plant development (e.g., root architecture, germination, senescence), all controlled by the four WRKY TFs, should also be taken into consideration.

## Data availability statement

The original contributions presented in the study are included in the article/**Supplementary Material**, further inquiries can be directed to the corresponding author/s.

## Author contributions

AK designed and supervised the study. AK, JR, AA-M, MB and KS performed the experiments. AK interpreted results and wrote the manuscript. All authors revised the manuscript.

## Supplementary material

Supplementary Figure 1 Selected Gene Ontology Terms for WRKY33, WRKY40, WRKY46, and WRKY75 enriched in the target gene set common to all four WRKY TFs. Bar lengths indicate -log_10_ -transformed False Discovery Rate (FDR). BP – Biological Process, MF – Molecular Function, CC – Cellular Component.

Supplementary Figure 2 Detailed localization of H_2_O_2_ accumulation in the cytoplasm in response to salinity. Staining of Arabidopsis leaves with an H_2_O_2_-specific BES-H_2_O_2_-Ac probe for intracellular hydrogen peroxide detection was carried out as described in Materials and Methods. Detection of BES-H_2_O_2_-Ac fluorescence is shown in false green **(A–F)**. The panel consists of images of leaves after 30 min of 150 mM NaCl applied directly to leaves **(A–C)** or 30 min of 250 mM NaCl applied directly to leaves **(D–F)**. Scale bar: 10 µm. This panel shows three examples of individual optical sections from the projections shown in **Figure 6II O**, **S**.

Supplementary Figure 3 Model presenting putative regulation of WRKYs expression by SnRK2.10 in response to salinity. Model is based on the results published by Sorenson et al. (2018); Soma et al. (2017); Kawa et al. (2020) and the present data. In response to salinity, SnRK2.10 is phosphorylated and interplays with cellular H_2_O_2_/ROS which triggers specific signaling pathways leading to the induction of expression of *WRKY33, WRKY40, WRKY46*, and *WRKY75.* Synthetized mRNA may be translated, which leads to the formation of WRKYs protein and specific regulation of stress response. Parallelly, SnRK2.10 may phosphorylate VCS and influence the 5’mRNA decay system, thus alternatively post-transcriptionally controlling WRKYs expression.

Supplementary Table 1 Primers used in this study.

Supplementary Data Sheet 1 Target genes of WRKY33, WRKY40, WRKY46, and WRKY75.

Supplementary Data Sheet 2 Protein partners of WRKY33, WRKY40, WRKY46, and WRKY75.
