# Supplementary figures and images for: SnRK2.10 kinase differentially modulates expression of hub WRKY transcription factors genes under salinity and oxidative stress in Arabidopsis thaliana
Source: Front Plant Sci. 2023 Aug 9;14:1135240. doi: 10.3389/fpls.2023.1135240 (PMC10445769; doi:10.3389/fpls.2023.1135240)

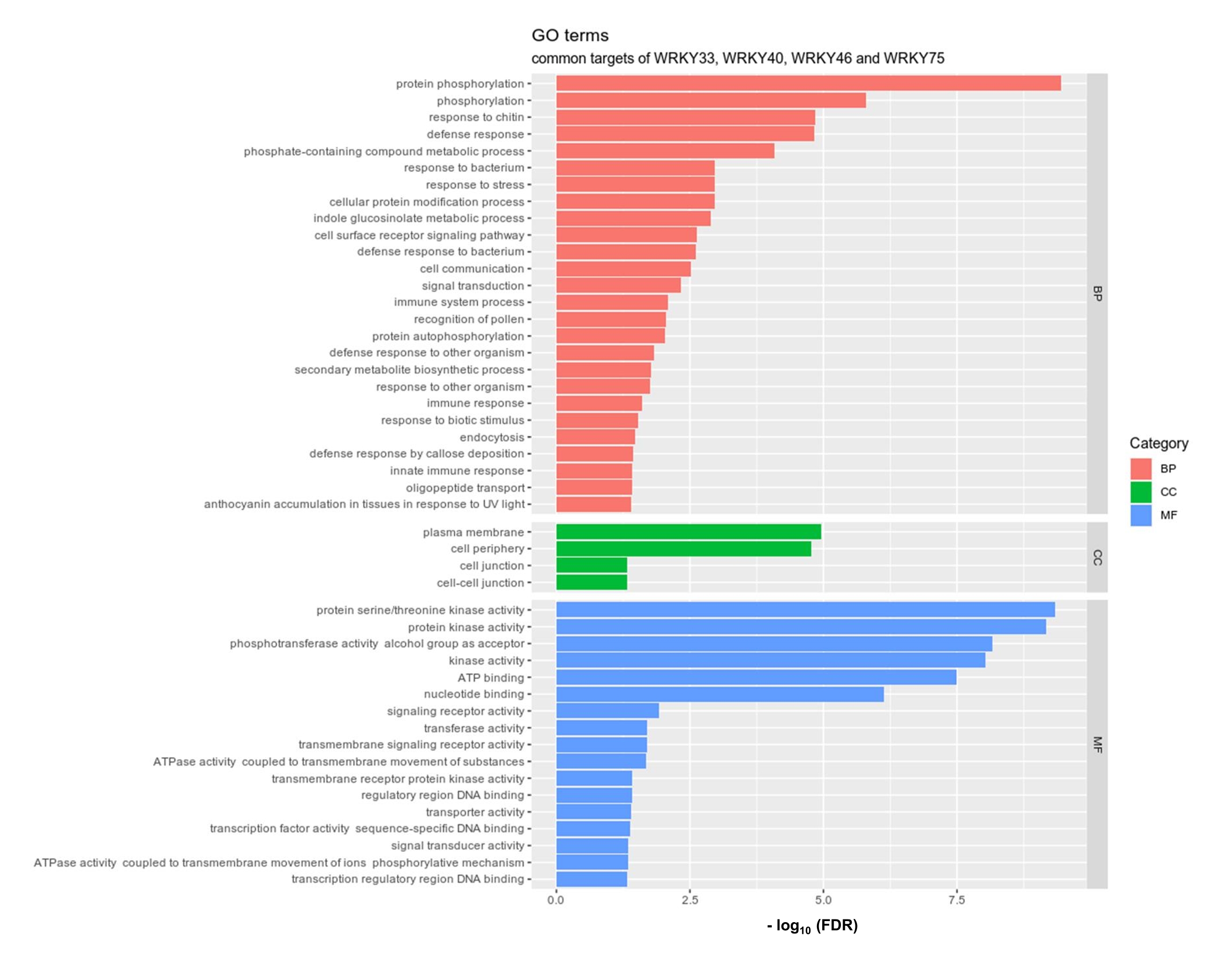

Supplement: Supplementary Figure 1 — Selected Gene Ontology Terms for WRKY33, WRKY40, WRKY46, and WRKY75 enriched in the target gene set common to all four WRKY TFs. Bar lengths indicate -log10 -transformed False Discovery Rate (FDR). BP – Biological Process, MF – Molecular Function, CC – Cellular Component. [file Image_1.jpeg]

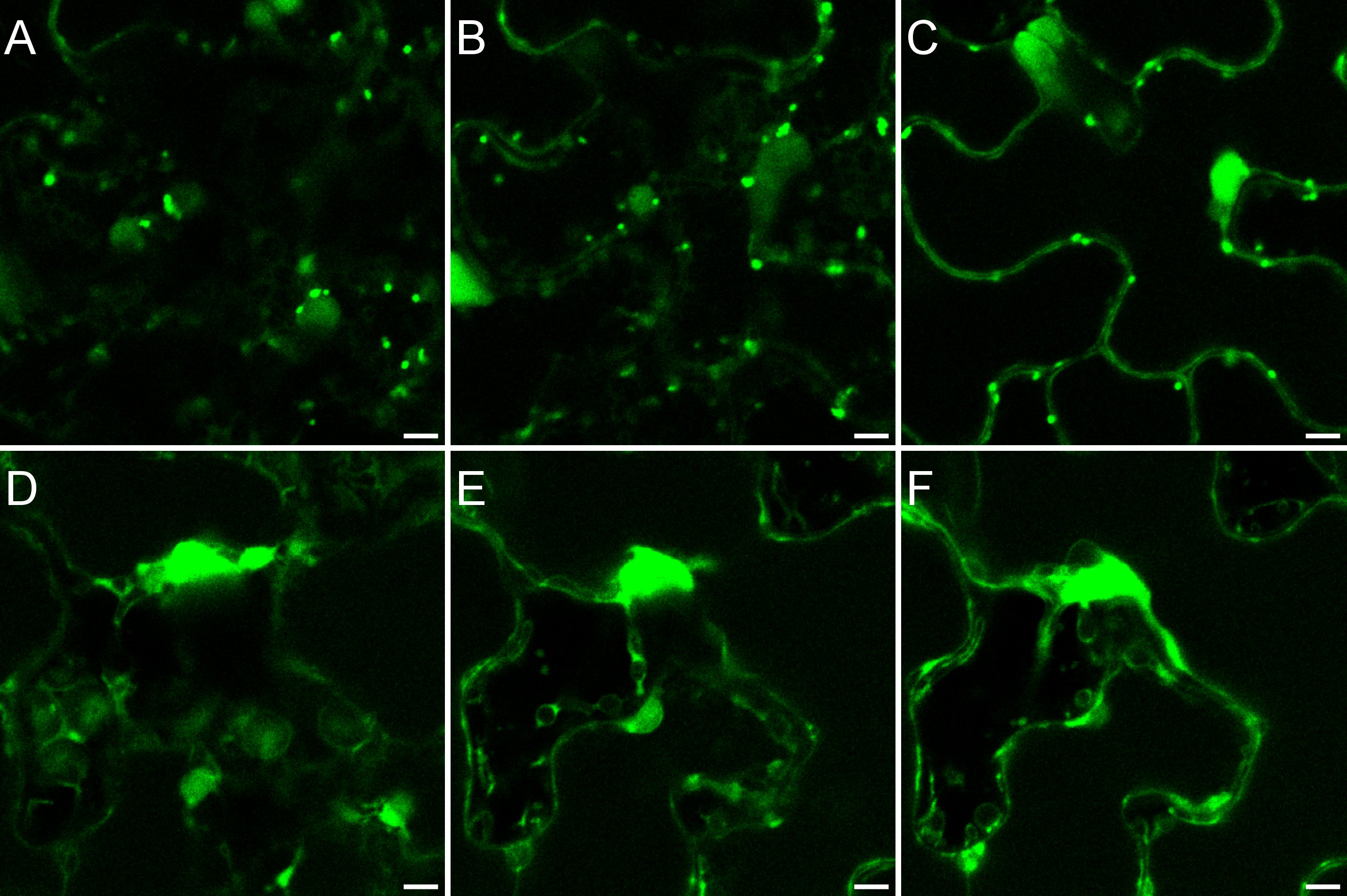

Supplement: Supplementary Figure 2 — Detailed localization of H2O2 accumulation in the cytoplasm in response to salinity. Staining of Arabidopsis leaves with an H2O2-specific BES-H2O2-Ac probe for intracellular hydrogen peroxide detection was carried out as described in Materials and Methods. Detection of BES-H2O2-Ac fluorescence is shown in false green (A–F). The panel consists of images of leaves after 30 min of 150 mM NaCl applied directly to leaves (A–C) or 30 min of 250 mM NaCl applied directly to leaves (D–F). Scale bar: 10 µm. This panel shows three examples of individual optical sections from the projections shown in Figure 6II O , S . [file Image_2.jpeg]

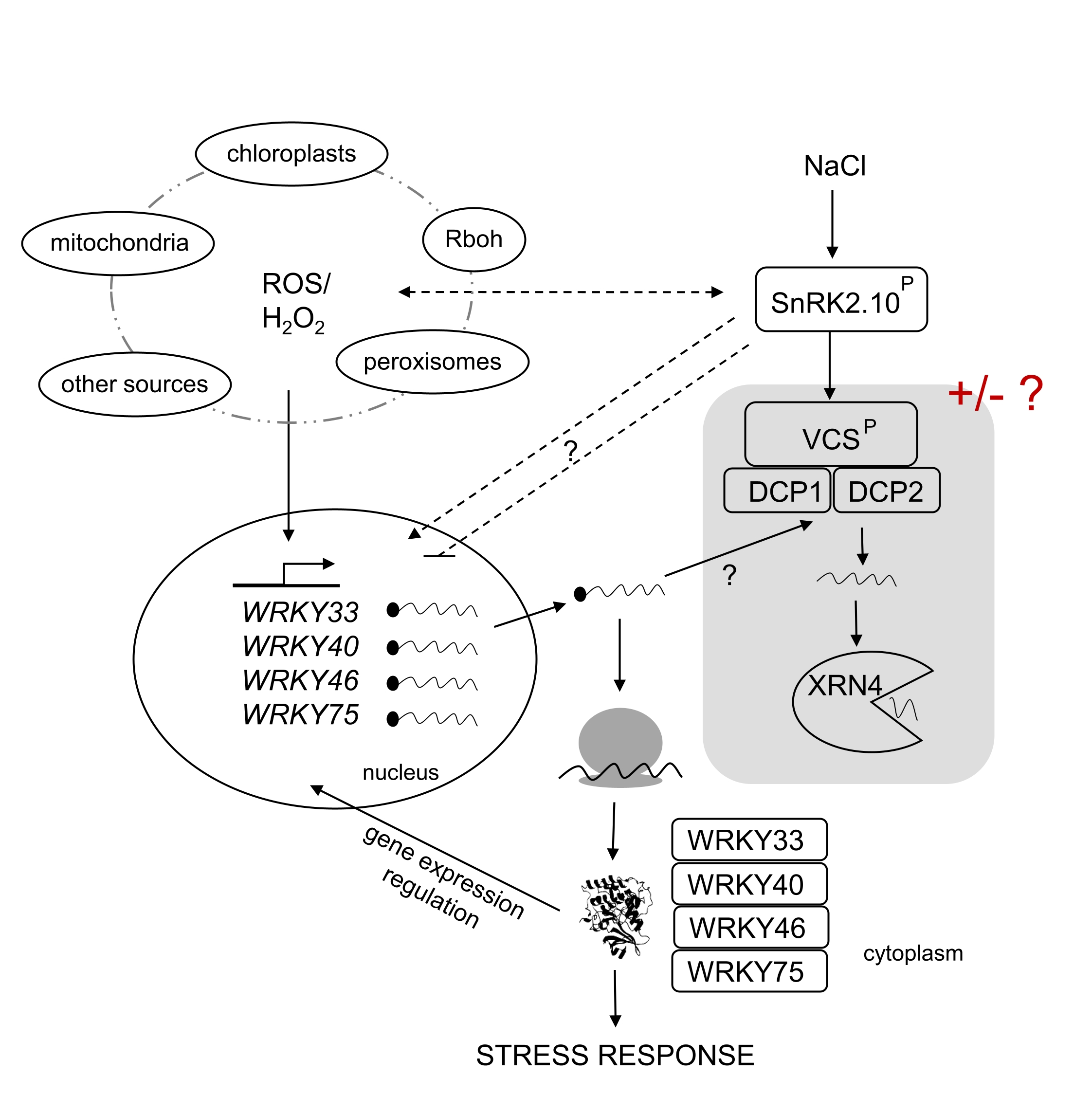

Supplement: Supplementary Figure 3 — Model presenting putative regulation of WRKYs expression by SnRK2.10 in response to salinity. Model is based on the results published by Sorenson et al. (2018); Soma et al. (2017); Kawa et al. (2020) and the present data. In response to salinity, SnRK2.10 is phosphorylated and interplays with cellular H2O2/ROS which triggers specific signaling pathways leading to the induction of expression of WRKY33, WRKY40, WRKY46, and WRKY75. Synthetized mRNA may be translated, which leads to the formation of WRKYs protein and specific regulation of stress response. Parallelly, SnRK2.10 may phosphorylate VCS and influence the 5’mRNA decay system, thus alternatively post-transcriptionally controlling WRKYs expression. [file Image_3.jpeg]
