# Supplementary material for: SnRK2.10 kinase differentially modulates expression of hub WRKY transcription factors genes under salinity and oxidative stress in Arabidopsis thaliana
Source: Front Plant Sci. 2023 Aug 9;14:1135240. doi: 10.3389/fpls.2023.1135240 (PMC10445769; doi:10.3389/fpls.2023.1135240)
Supplement: Supplementary Table 1 — Primers used in this study. [file Table_1.docx]

| **Table S1. List of primers used in this study** | | |
| --- | --- | --- |
|  |  |  |
| AGI | Gene name | Primers |
| AT1G60940 | *SNRK2.10* | F -TGGAGTGAGAATGTCGGGTTCT |
|  |  | R - TCCCGTATATTTGTCACCAACTCG |
| AT1G13320 | *PDF2* | F - TAACGTGGCCAAAATGATGC |
|  |  | R - GTTCTCCACAACCGCTTGGT |
| AT5G25760 | *PEX4* | F - CTGCGACTCAGGGAATCTTCTAA |
|  |  | R - TTGTGCCATTGAATTGAACCC |
| AT2G38470 | *WRKY33* | F - CTCGTGGTAGCGGTTACGCC |
|  |  | R - CCTTTGCCTCTAGAGAATCCACC |
| AT1G80840 | *WRKY40* | F - AAATCAGCCCTCCCAAGAAACG |
|  |  | R - CTTCACGACAGTCTCTTCTCTCTGC |
| AT2G46400 | *WRKY46* | F- ACATCACATCCCCGAAGACG |
|  |  | R - ACTTCTTCGGACTTGGTCGG |
| AT5G13080 | *WRKY75* | F - AGGCCGTCAAGAACAACAAG |
|  |  | R - ACGACCACTTCTTGGTCCAC |
| AT4G11650 | *OSM34* | F - ACGGTCAGGGATCATGTAGCG |
|  |  | R - TAGCTGTAAGCGTCAGGGCATC |
| AT2G30770 | *CYP71A13* | F - GTGCTTCGGTTGCATCCTTCTC |
|  |  | R - CGCCCAAGCATTGATTATCACCTC |
| AT4G27410 | *RD26* | F - GAAGGTGAGGCGGAGAGTG |
|  |  | R - CCCGAAACTCTGAGTCAACCT |
| AT1G01720 | *ATAF1* | F - GCCTCTCGGTAGCTCCTTTTTTGT |
|  |  | R - TCAGGCTGGATGATTGGGTTCTCT |
| AT5G04340 | *ZAT6* | F - GCCTCCGTTCTTTCCTTCGTAGTG |
|  |  | R - TGTGAAGTCGCACGTTTGCTCTAT |
